# Comparative analysis of infective endocarditis in hemodialysis versus non-hemodialysis patients in Iran: implications for clinical practice and future research

**DOI:** 10.1186/s12872-023-03675-0

**Published:** 2024-01-02

**Authors:** Fereshteh Zolfaghari, Mohammad Mehdi Peighambari, Erfan Kohansal, Anita Sadeghpour, Pardis Moradnejad, Zahra Shafii

**Affiliations:** 1 Rajaie Cardiovascular Medical and Research Center, Iran University of Medical Sciences, Vali- Asr Ave, Tehran, 1995614331 Iran; 2 Cardiovascular Intervention Research Center, Rajaie Cardiovascular Medical and Research Center, University of Medical Sciences, Tehran, Iran; 3 MedStar Health Research Institute, Washington, DC USA

**Keywords:** Infective endocarditis, Hemodialysis, Clinical characteristics, Prognosis

## Abstract

**Background:**

In hemodialysis (HD) patients, there is a larger frequency of mortality and morbidity associated with infective endocarditis (IE) as opposed to the general population. Despite the increased burden of IE in the HD population, optimal strategies for prevention and management still need to be clarified. Elucidating the distinguishing features and outcomes of IE in HD patients is crucial to guide clinical decision-making and improve prognosis in this high-risk group. However, the details of IE characteristics, specifically in HD patients in the Middle East, are limited.

**Objective:**

To compare the clinical characteristics and short-term outcomes of IE between HD and non-HD patients.

**Methods:**

A retrospective analysis was carried out on 139 patients with infective endocarditis who were referred to a tertiary cardiovascular center in Iran from 2006 to 2018. The participants were split into HD (n = 34) and non-HD (n = 105) groups. Data pertaining to demographic characteristics, comorbidities, microbiological findings, occurrence of complications, therapeutic interventions, and mortality rates during hospital stay were gathered.

**Results:**

Diabetes, hypertension, and congestive heart failure were observed more frequently in HD patients. HD patients were more likely than non-HD patients to have involvement of the right valve (41.2% vs. 20.9%), larger vegetation, and extracardiac emboli. In-hospital mortality was 41.2% for HD patients versus 14.3% for non-HD patients. Mortality remained high after valve surgery in HD patients (38.2% vs. 10.5% in non-HD).

**Conclusion:**

HD patients exhibited a distinct clinical profile of IE with worse short-term outcomes, including higher mortality.

## Introduction

Despite advancements in diagnostic tools and medicine, infective endocarditis (IE) remains a life-threatening condition correlated with high morbidity and mortality [1]. IE affects approximately 3–10 individuals per 100,000 annually in the general population [2]. However, certain groups have been shown to be at substantially increased risk of IE. End-stage renal disease (ESRD) patients represent one such high-risk group [2].

According to estimates, the prevalence of IE in chronic hemodialysis patients is 50–180 times higher than that in the general population [3, 4]. Thus, they have higher morbidity and mortality rates [1, 5]. In a large study examining IE in hemodialysis patients, Abbott and Agodoa found 267 cases per 100,000 patient-years, almost 100 times higher than that in the general population [6]. According to a recent meta-analysis, the prevalence of IE in patients with chronic HD was 2.7 − 3.1%, with in-hospital and long-term mortality rates of 29.5% and 45.6%, respectively [7].

This group’s high incidence of IE can be attributed to various factors [8]. Disturbed calcium and phosphate homeostasis in individuals undergoing HD can lead to premature onset and an increased prevalence of degenerative valvular abnormalities and calcification [8]. Moreover, recurrent bacteremia due to regularly occurring vascular instrumentation through vascular grafts or catheters and uremia-related compromised immune systems collectively predispose HD patients to IE [8–10].

In addition to the incidence of IE, the microbiology and clinical profiles of patients undergoing hemodialysis differ from those of patients with IE in the general population. Staphylococcus aureus is the predominant causative organism, accounting for 37–65% of cases [8, 11–13]. This is in contrast to the general population, where streptococci remain the leading cause of IE [14]. The frequent vascular punctures required for access are probably responsible for high prevalence of S. aureus. Right-sided IE is also more common, representing up to 58% of cases [11]. This compares to only 5–10% in the general population [15].

In recent decades, there has been a major change in the epidemiology of IE [8]. The increased prevalence of diabetes and hypertension, with the consequent rise in end-stage renal disease incidence, requires sufficient attention to IE and its outcome [11, 16]. Furthermore, HD patients suffering from IE have a poor prognosis, and therapeutic advances have failed to improve survival rates over the past two decades [16, 17].

Despite the well-established increased burden of infective endocarditis (IE) in hemodialysis (HD) patients, many knowledge gaps remain regarding optimal prevention and treatment approaches for this vulnerable group. Mortality rates have persistently remained high, ranging from 29.5 to 45.6% in recent studies, indicating that current management strategies are inadequate [5, 18–20]. Further research is critically required to clarify best practices and improve the poor prognosis of IE in HD patients. In particular, data elucidating the distinctive clinical and microbiological characteristics of IE in the HD population still needs to be included, especially in Middle Eastern cohorts.

To the best of our knowledge, there is limited information describing the features and outcomes of IE, specifically in Iranian HD patients. Therefore, we aimed to conduct a detailed comparison of the clinical and microbiological profile, treatment patterns, and short-term mortality of IE between HD patients and non-HD patients admitted to a tertiary cardiac center in Tehran, Iran. Defining the epidemiology and prognosis of IE in this high-risk group will inform prevention and management strategies.

## Methods

### Study design and population selection

This retrospective study used data from the Iranian Registry of Infective Endocarditis (IRIE), which collects information on all IE admissions to Rajaie Cardiovascular Medical and Research Center, a tertiary referral center in Tehran, Iran. Patients were identified by searching the IRIE database for all admissions from January 2006 to December 2018 with a final diagnosis of definite or possible IE per the modified Duke criteria [21].

The IRIE contains detailed information on demographics, presenting symptoms, comorbidities, predisposing factors, microbiological data, echocardiographic findings, treatment, and outcomes for each patient. Data accuracy in the IRIE is preserved through quality control measures, including oversight by attentive personnel and regular database monitoring [22]. The exclusion criteria were age less than 16 years, outpatient treatment only, and if an IE diagnosis was ruled out post-hospitalization. The study protocol conforms to the ethical guidelines of the 1975 Declaration of Helsinki and was reviewed and approved by the Institutional Ethics Committee (IR.IUMS.FMD.REC.1398.495).

As mentioned earlier, the study sample was derived from the IRIE, which contained records for 602 IE admissions to our cardiovascular center. Of these, 34 patients had documented hemodialysis status prior to IE hospitalization. These 34 HD cases represented the primary cohort of interest.

The remaining 568 non-HD patients were considered as potential controls. To generate an appropriately sized unbiased control group for comparison, we utilized simple random sampling. Specifically, the 568 non-HD cases were assigned computer-generated random number IDs. We then selected the first 105 non-HD cases based on the randomized numbers, representing approximately triple (3x) the size of the HD group. This control ratio helps provide statistical power while limiting skewing the analysis heavily in favor of the larger subgroup.

As the non-HD controls were randomly selected from the same institutional IE admissions, major demographic and clinical characteristics were comparable between groups at baseline, with the main differentiator being HD status. This sampling methodology aimed to generate an unbiased, representative control cohort suitable for contrasting key outcomes of interest against the primary HD population.

Data were collected from medical records on demographics, comorbidities, predisposing cardiac conditions, dialysis details (for the HD group), presumed source of infection, causative organisms, laboratory results, echocardiographic findings, complications, treatment modalities, and in-hospital mortality.

Comorbidities and predisposing cardiac conditions assessed included cerebrovascular diseases, diabetes mellitus, hypertension, prior IE, IV drug abuse, use of immunosuppressive agents, rheumatic diseases, valvular heart diseases, congestive heart failure, prior cardiac surgeries, congenital heart diseases, and degenerative valve diseases.

Transesophageal echocardiography was employed to evaluate vegetation size and location, cardiac function indices (such as left ventricular ejection fraction), and any structural complications, including perivalvular abscesses, valve perforations, pseudoaneurysms, intracardiac fistulae, tamponade, and prosthetic valve malfunction. In addition, extracardiac complications such as abscess formation and emboli to vital organs were documented. The early outcomes evaluated were in-hospital mortality and length of stay.

### Statistical analysis

Descriptive statistics were used to summarize the demographic and clinical characteristics. Continuous variables are reported as mean ± standard deviation for normally distributed data or median (interquartile range) for skewed data. Categorical variables are reported as numbers (percentages). Comparisons between the HD and non-HD groups were made using the chi-square test for categorical data. For continuous data, normality was assessed using the Shapiro-Wilk test. If the data were normally distributed, an independent sample t-test was used between groups. For non-normally distributed continuous variables, the Mann–Whitney U test was applied.

All statistical tests were two-sided, and a *p*-value < 0.05 was considered statistically significant. Analyses were performed using SPSS version 25.0 (IBM, Armonk, NY). The number of IE admissions recorded in the IRIE registry between January 2006 and December 2018 determined the sample size.

## Results

### Demographics

There were no significant differences in gender (67.65% male in HD [n = 23] vs. 62.86% in non-HD [n = 66]; *P* = 0.613) or mean age (49.85 ± 18.139 years in HD vs. 45.30 ± 17.4 years in non-HD; *P* = 0.192) between the groups.

The majority of patients, both in the HD and non-HD groups, had a definite diagnosis of IE based on modified Duke criteria, with no significant difference between the groups (73.53% of HD patients [n = 25] and 75.24% of non-HD patients [n = 79]; *P* = 0.842). In addition, possible IE was diagnosed in nearly one-quarter of both groups (26.47% of HD [n = 9] and 24.76% of non-HD [n = 26]; *P* = 0.842). Taken together, these findings demonstrate that HD and non-HD patients had similar rates of definite and possible IE according to the diagnostic criteria.

With respect to IE onset, acute presentations predominated in both groups without significant differences between HD and non-HD patients (58.82% [n = 20] vs. 56.19% [n = 59]; *P* = 788). Subacute IE occurred at low and comparable frequencies (5.88% of HD [n = 2] and 18.10% of non-HD [n = 19]; *P* = 0.084). Notably, compared with non-HD patients, chronic IE was substantially more prevalent in HD patients (8.82% [n = 3] vs. 0.95% [n = 1]; *P* = 0.017).

In the HD group, 14.71% (n = 5) had arteriovenous fistulae, 79.41% (n = 27) had central venous catheters, and 5.88% (n = 2) had both as dialysis access. Six patients (17.65%) had a history of kidney transplant rejection. The mean dialysis duration before IE admission was 33.8 ± 33.087 months.

### Comorbidities and predisposing conditions

The prevalence of several key comorbidities differed between the group (Table 1). Diabetes mellitus was identified in 38.2% (n = 13) of HD patients compared with 16.2% (n = 17) of non-HD patients. Hypertension affected 61.8% (n = 21) of HD patients and 17.1% (n = 18) of non-HD patients. Congestive heart failure was also more common in the HD group at 29.4% (n = 10) versus 13.3% (n = 14) in non-HD patients. Previous history of IE, cerebrovascular disease, intravenous drug use, and immunosuppressant use did not significantly differ between the groups.

**Table 1:** Patients baseline characteristics

	HD Group (n = 34)	Non-HD Group (n = 105)	*P*-value
**Age** (years) — Mean ± SD	49.85 ± 18.139	45.30 ± 17.4	*0.560*
**Male** — No. (%)	23 (67.65%)	66 (62.86%)	*0.613*
**Dialysis Access Type** — No. (%)			
Arteriovenous fistulae	5 (14.71%)	-	*-*
Central venous catheters	27 (79.41%)	-	*-*
Both	2 (5.88%)	-	*-*
**Infective Endocarditis Diagnosis** — No. (%)			
Definite	25 (73.53%)	79 (75.24%)	*0.842*
Acute	20 (58.82%)	59 (56.19%)	*0.788*
Subacute	2 (5.88%)	19 (18.10%)	*0.084*
Chronic	3 (8.82%)	1 (0.95%)	***0.017***
Possible	9 (26.47%)	26 (24.76%)	*0.842*
**Past Medical History & Comorbidities** — No. (%)			
Cerebrovascular Diseases	5 (14.71%)	7 (6.66%)	*0.147*
Diabetes Mellitus	13 (38.23%)	17 (16.19%)	***0.023***
Hypertension	21 (61.76%)	18 (17.14%)	***0.001***
Prior IE	7 (20.58%)	10 (9.52%)	*0.087*
IV Drug Abuse	1 (2.94%)	10 (9.52%)	*0.217*
Use of Immunosuppressive Agents	4 (11.76%)	4 (3.80%)	*0.083*
Rheumatic Heart Diseases	0 (0.0%)	5 (4.76%)	*0.195*
Non-rheumatic Valvular Heart Diseases	21 (61.76%)	72 (68.57%)	*0.464*
Aortic Regurgitation	7 (20.58%)	10 (6.66%)	*0.087*
Aortic Stenosis	1 (2.94%)	3 (2.85%)	*0.980*
Mitral Regurgitation	11 (32.35%)	25 (23.80%)	*0.323*
Mitral Stenosis	0 (0.0%)	4 (3.80%)	*0.248*
Tricuspid Regurgitation	14 (41.17%)	51(48.57%)	*0.453*
Tricuspid Stenosis	1 (2.94%)	1 (0.95%)	*0397*
Pulmonary Regurgitation	5 (14.70%)	26 (24.76%)	*0.212*
Pulmonary Stenosis	0 (0.0%)	2 (1.90%)	*0.568*
Congestive Heart Failure	10 (29.41%)	14 (13.33%)	***0.033***
History of Cardiac Valve Surgeries	5 (14.7%)	30 (28.57%)	*0.105*
History of CABG	3 (8.82%)	2 (1.90%)	*0.600*
Congenital Heart Diseases	8 (23.52%)	38 (36.19%)	*0.843*
Degenerative Valve Diseases	1 (2.94%)	8 (7.61%)	*0.864*

The frequency of prior cardiac valve surgery was 14.71% (n = 5) in HD and 28.57% (n = 30) in non-HD patients, with mitral valve surgery being the most common in HD and aortic valve surgery being the most common in non-HD. Mechanical prosthetic valves were present in 5.88% (n = 2) of HD patients and 19.05% (n = 20) of non-HD patients.

The most common structural heart disease was non-rheumatic valve disease in both groups (Table 1). Subsequently, congenital heart defects were most frequent, including bicuspid aortic valve (11.76%, n = 4) and ventricular septal defect (5.88%, n = 2) in HD and bicuspid aortic valve (12.38%, n = 13) and mitral valve prolapse (7.62%, n = 8) in the non-HD group.

### Microbiological profile

Positive blood cultures occurred in 50% (n = 17) of HD patients and 54.29% (n = 57) of non-HD patients (*P* = 0.663). The most frequent responsible pathogens were coagulase-negative staphylococci (14.71%, n = 5) and Staphylococcus aureus (11.76%, n = 4) in the HD group, and Staphylococcus aureus (14.29%, n = 15) and enterococci (10.48%, n = 11) in the non-HD group. Methicillin-resistant Staphylococcus aureus (MRSA) prevalence was similar between the HD and non-HD groups (5.88% of the HD group [n = 2] and 4.76% of the non-HD group [n = 5]; *P* = 0.681) (Table 2). Although not statistically significant, the percentage of fungal infections (including Candida and Aspergillus) was higher in the HD group than in the non-HD group (8.82% of the HD group [n = 3] and 1.9% of the non-HD group [n = 2]; *P* = 0.094).

**Table 2:** Comparison of microbiological findings between the HD and non-HD patients with IE

Micro-organisms	HD Group (n = 34)		Non-HD Group (n = 105)		*P*-value
S. aureus	4	(11.76%)	13	(12.38%)	*0.910*
MRSA	2	(5.88%)	5	(4.76%)	*0.681*
Coagulase Negative Staphylococci	5	(14.71%)	9	(8.57%)	*0.234*
Enterococci	3	(8.82%)	11	(10.48%)	*0.781*
S. viridans	1	(2.94%)	6	(5.71%)	*0.520*
Streptococcus Alpha-hemolytic	0		5	(4.76%)	*0.240*
Fungal infection	3	(8.82%)	2	(1.90%)	*0.094*
Brucella	0		4	(3.81%)	*0.321*
Pseudomonas	1	(2.94%)	3	(2.86%)	*0.679*
gram Negative Bacillus	1	(2.94%)	3	(2.86%)	*0.679*
Klebsiella	1	(2.94%)	2	(1.90%)	*0.572*
Acinetobacter	1	(2.94%)	2	(1.90%)	*0.572*
Coxiella burnetti	0		2	(1.90%)	*0.569*
S. bovis	0		1	(0.95%)	*0.755*
E. coli	0		1	(0.95%)	*0.755*
Serratia marcescens	0		1	(0.95%)	*0.755*

### Laboratory findings

Serum hemoglobin levels were significantly lower in the HD group than in the non-HD group. Other laboratory findings are depicted in Table 3.

**Table 3:** Comparison of laboratory findings between the HD and non-HD patients with IE

Average of Laboratory Findings	HD Group (n = 34)	Non-HD Group (n = 105)	*P*-value
Serum WBC count, /mm3	9889.7 ± 5035.4	9633.3 ± 4403.3	*0.776*
Serum hemoglobin, g/dL	9.1 ± 1.8	10.4 ± 2.2	***0.003***
ESR, mm/h	54.4 ± 27.5	49.8 ± 28.5	*0.421*
CRP, mg/L	59.1 ± 49.4	48.5 ± 42.2	*0.275*

HD, Hemodialysis; IE, Infective endocarditis; WBC, White blood cell; ESR, Erythrocyte sedimentation rate; CRP, C-reactive protein

### Echocardiographic findings

The most common vegetation site was left-sided valves in both groups, with no statistically significant difference (64.71% in the HD group [n = 22] vs. 75.24% in the non-HD group [n = 79]). No statistically considerable difference was observed between the two groups in terms of vegetation locations except for right-sided valves, which were significantly more affected in HD patients than non-HD patients (41.18%, [n = 14] vs. 20.95%, [n = 22]; *P* = 0.019), especially the tricuspid valve (38.24%, [n = 13] vs. 14.29%, [n = 15]). Simultaneous right- and left-sided vegetation was also more common in HD patients (14.71%, [n = 5] vs. 3.81%, [n = 4]; *P* = 0.025). The mean vegetation size was significantly larger in HD patients than in non-HD patients (18.6 ± 11.8 mm vs. 12.1 ± 8.7 mm; *P* < 0.001). Vegetation on the dialysis catheter was reported in 11.76% (n = 4) of HD patients. There was no significant difference in prosthetic valve involvement between the two groups. Vegetation sites in HD and non-HD patients with IE are shown in Table 4.

**Table 4:** Vegetation sites in the HD and non-HD patients with IE

Vegetation Sites	HD Group (n = 34)	Non-HD Group (n = 105)	*P*-value
Left-sided native and prosthetic valve vegetation	22 (64.70%)	79 (75.23%)	*0.231*
Left-sided native valve vegetation	20 (58.82%)	61 (58.09%)	*0.940*
Left-sided prosthetic valve vegetation	2 (5.88%)	20 (19.04%)	*0.068*
Right-sided native and prosthetic valve vegetation	14 (41.17%)	22 (20.95%)	***0.019***
Right-sided native valve vegetation	14 (41.17%)	19 (18.09%)	***0.01***
Right-sided prosthetic valve vegetation	0	4 (3.80%)	*0248*
Both-sided native and prosthetic valve vegetation	5 (14.7%)	4 (3.80%)	***0.025***
AV site vegetation	12 (35.29%)	42 (40%)	*0.625*
MV site vegetation	14 (41.17%)	47 (44.76%)	*0.714*
TV site vegetation	13 (38.23%)	15 (14.28%)	***0.002***
PV site vegetation	2 (5.88%)	8 (7.61%)	*0.733*

HD, Hemodialysis; IE, Infective endocarditis; AV, Aortic valve; MV, Mitral valve; TV, Tricuspid valve; PV, Pulmonary valve

### Outcomes and course of the disease

The incidence rates of IE-related intracardiac complications are presented in Table 5. Conduction abnormalities were the only intracardiac complications that were significantly higher in the HD group. Although the percentage of prosthetic paravalvular leakage was higher in the non-HD group than in the HD group, the difference failed to constitute statistical significance.

**Table 5:** Cardiac complications in the HD and non-HD patients with IE

Cardiac Complications	HD Group (n = 34)	Non-HD Group (n = 105)	*P*-value
Cardiac fistulae	0	3 (2.85%)	*0.319*
Perivalvular abscesses	2 (5.88%)	11 (10.47%)	*0.424*
Valve perforations	5 (14.70%)	25 (23.80%)	*0.262*
Dehiscence	0	3 (2.85%)	*0.319*
Prosthetic paravalvular leakage	0	10 (9.52%)	*0.062*
Intervalvular fibrosa pseudoaneurysms	3 (8.82%)	9 (8.57%)	*0.964*
Heart failure	19 (55.88%)	41 (39.04%)	*0.085*
Tamponade	1 (2.94%)	5 (4.76%)	*0.758*
Acute myocardial infarction	0	0	*-*
ECG abnormalities	3 (8.82%)	1 (0.95%)	***0.017***

HD, Hemodialysis; IE, Infective endocarditis

Concerning IE-related extra-cardiac complications, although extracardiac abscess rates were similar between groups, the HD cohort had a significantly higher incidence of extra-cardiac embolic events compared with non-HD patients (44.12% [n = 15] vs. 21.90% [n = 23]). Pulmonary artery embolism was the most frequent embolic complication in the HD group. Despite increased embolic risk, rates of acute cerebrovascular events such as ischemic stroke, hemorrhage, and mycotic aneurysms were not significantly different between the two groups. (Table 6)

**Table 6:** Extra-cardiac complications in the HD and non-HD patients with IE

Extra-cardiac Complications	HD Group (n = 34)	Non- HD Group (n = 105)	*P*-value
Extra-cardiac abscesses	7 (20.58%)	11 (10.47%)	*0.127*
Splenic abscesses	2 (5.88%)	6 (5.71%)	*0.971*
Brain abscesses	1 (2.94%)	1 (0.95%)	*0.397*
Lung abscesses	4 (11.76%)	5 (4.76%)	*0.154*
Extra-cardiac embolic events	15 (44.11%)	23 (21.90%)	***0.027***
Cerebral emboli	2 (5.88%)	8 (7.61%)	*0.724*
Pulmonary emboli	10 (29.41%)	14 (13.33%)	***0.031***
Splenic emboli	0	1 (0.95%)	*0.568*

HD, Hemodialysis; IE, Infective endocarditis

The most commonly administered antibiotics were vancomycin (85.29%, n = 29), meropenem (55.88%, n = 19), ciprofloxacin (29.41%, n = 10), linezolid (17.65%, n = 6), and ceftriaxone (17.65%, n = 6) in the HD group, and vancomycin (70.48%, n = 74), gentamicin (50.48%, n = 53), ampicillin (31.43%, n = 33), and ceftriaxone (25.71%, n = 27) in the non- HD group.

In-hospital mortality was noticeably higher in the HD group than in the non-HD group (41.18% [n = 14] vs. 14.29% [n = 15]). The most common cause of death in both groups was sepsis-induced multi-organ failure.

Overall, 18 patients (52.94%) in the HD group and 60 (57.14%) in the non-HD group underwent valve surgeries. As demonstrated in Table 7, a significantly higher proportion of HD patients died during their course of hospitalization compared to non-HD patients among those who underwent surgical intervention (38.9% [n = 7] vs. 10% [n = 6]; *P* = 0.008). Although survival rates were numerically higher in medically treated non-HD patients compared to HD patients (80% [n = 36] vs. 56.3% [n = 9]), this difference did not reach statistical significance (*P* = 0.067). Thus, survival outcomes were similar between medically managed HD and non-HD groups.

**Table 7:** Therapeutic interventions and survival outcomes in the HD and non-HD patients with IE

Interventions and Outcomes	HD Group (n = 34)	Non-HD Group (n = 105)	*P*-value
Medical intervention	16 (47.1%)	45 (42.9%)	*0.668*
Survived	9 (56.3%)	36 (80%)	*0.067*
Dead	7 (43.8%)	9 (20%)	
Surgical Intervention	18 (52.9%)	60 (57.1%)	*0.668*
Survived	11 (61.1%)	54 (90%)	***0.008***
Dead	7 (38.9%)	6 (10%)	

HD, Hemodialysis; IE, Infective endocarditis

Among the subgroup of HD patients, there was no significant difference in in-hospital mortality rates when comparing those treated medically to those undergoing surgical intervention (43.8% [n = 7] vs. 38.9% [n = 7]; *P* = 0.773).

The mean length of hospital stay was 29.65 ± 17.21 days in the HD group and 37.03 ± 21.17 days in the non-HD group. Although the length of hospital stay was longer in the non-HD group, the difference was not statistically significant (*P* = 0.067). Seventy-five patients required intensive care unit admission (47.6% [n = 16] in the HD group vs. 56.19% [n = 59] in the non-HD group; *P* = 0.353). Moreover, the mean length of ICU stay did not significantly differ between the two groups (7.88 ± 6.52 vs. 5.24 ± 3.659 days; *P* = 0.056).

## Discussion

### Demographics

Male patients comprised the majority of our study population in both HD and non-HD groups. Gender differences are reported heterogeneously in other studies. Pericas et al. and Kwon et al. found predominantly female hemodialysis IE patients, whereas Bentata et al., Stahl et al., and Zhang et al. noted mostly males in both groups [19, 20, 23–25]. This may be due to regional differences in the cohorts.

In the present study, the mean ages of HD and non-HD patients were not significantly different, which is in line with studies by Bentata et al., Kwon et al., Hsiao et al., and Zhang et al., who have not noted any statistically significant age difference between the groups [20, 23, 24, 26]. In contrast, Stahl et al. and Bhatia et al. reported significantly lower mean ages in the hemodialysis group [8, 25]. This diversity is likely due to the smaller number of older hemodialysis patients in their cohorts. However, our numbers are noticeably lower than those reported in a review of other studies on the epidemiological features of IE. The disparity mentioned earlier can be an important issue for future research.

Our results highlight chronic IE as a potential concern in the HD population. Overall, the IE onset types indicate that dialysis status does not overtly alter the typical acute presentation but may confer a higher risk for indolent chronic infection.

### Comorbidities and predisposing conditions

In this study, the prevalence rates of diabetes mellitus, hypertension, and congestive heart failure were significantly higher in the HD group than in the non-HD group. This finding is in accordance with prior epidemiological observations by Stahl et al., who reported diabetes mellitus, ischemic heart disease, and hypertension as significantly prevalent comorbidities in HD patients [25]. Our observations did not show any meaningful differences in the prevalence of cerebrovascular disease, intravenous drug use, history of cardiac valve surgeries, or immunosuppressant use between the study groups, contrary to some previous studies. For instance, Pericas et al. found that peripheral vascular disease and cerebrovascular disease were notably higher in the HD-IE group. However, intravenous drug use and congenital heart disease were more frequently found in the non-HD-IE group [19]. Stahl et al. reported that prosthetic heart valves prior to IE were significantly more common in the non-HD group [25]. Zhang et al. and Wang et al. consistently reported that predisposing cardiac anomalies were more frequently observed in the non-HD-IE group than in the HD-IE group [20, 27]. Accordingly, Bhatia et al. found a lower prevalence of most IE risk factors, including congenital heart disease, rheumatic heart disease, valvular heart disease, prior valve replacement, and history of drug abuse in dialysis patients [8].

In contrast to previous studies that reported rheumatic valve diseases as a notable risk factor for IE, only 4% of our studied patients suffered from such diseases, with no statistically significant difference between the HD and non-HD groups [28, 29]. Our study’s low percentage of rheumatic valve diseases could be because we considered only echocardiographic findings and did not analyze pathological findings. Furthermore, valve destruction caused by IE might mask rheumatic changes and render them undetectable by echocardiography.

The most common congenital heart defect in the HD group was BAV. While the prevalence of BAV in the general population is 1–2%, it was approximately 11.7% in our HD group [30]. Zegri-Reiriz et al. and Zhu et al. reported a higher risk of IE in patients with BAV and recommended antibiotic prophylaxis [31, 32].

### Microbiological profile

Negative blood cultures occurred in 50% of HD patients and 54.3% of non-HD patients. Similarly, Bentata et al. and Zhang et al. reported negative blood cultures in nearly half of their study population [20, 23]. These results differ from Stahl’s 2023 study, which reported 7.1% and 19.4% negative blood cultures in HD and non-HD patients with IE, respectively [25]. In the studies by Wang et al. and Hsiao et al., the reported numbers for positive cultures were over 70%. This discrepancy could be partially attributable to referral bias because, patients are often transferred to our tertiary center after antibiotic initiation [26, 27].

The current study found that coagulase-negative staphylococci were the most common pathogen, with Staphylococcus aureus being the second most common pathogen in the HD group. In the non-HD group, Staphylococcus aureus and enterococci were the most common and second-most common pathogens, respectively. The incidence of methicillin-resistant Staphylococcus aureus (MRSA) was observed to be similar across the various cohorts. On the contrary, almost all previous studies have documented Staphylococcus aureus as the prevailing causative pathogen responsible for endocarditis in individuals undergoing dialysis [8, 12, 18, 33–35]. Pericas et al. reported that the viridans group streptococci and Streptococcus bovis were found to be more commonly observed among non-hemodialysis patients, which is quite unexpected [19]. The epidemiological variability of infective endocarditis (IE) and the lack of research in Iran necessitate a comprehensive examination of antibiotic coverage for coagulase-negative staphylococci. Our HD cohort comprised of a limited patient population. Consequently, conducting future prospective investigations is warranted to assess microbiological characteristics more broadly.

### Laboratory findings

In accordance with the study by Zhang et al.’s, our investigations showed that the serum hemoglobin level was significantly lower in the HD group than in the non-HD group [20]. In addition, white blood cell (WBC), erythrocyte sedimentation rate (ESR), and C-reactive protein (CRP) levels were not significantly different between our studied groups. In contrast to our finding, investigations by Hsiao et al. showed that while CRP levels did not significantly differ between the hemodialysis and non-hemodialysis groups, ESR was markedly higher in the hemodialysis patients than in the non-HD patients [26].

### Echocardiographic findings

This study showed that the mitral valve is the most frequently involved cardiac valve, followed by the aortic valve, in all patients with IE. The aforementioned dominance remains unchanged in both HD and non-HD patients. This finding is backed up by pieces of evidence from most previous observations by Rekik et al., Nori et al., Zhang et al., Doulton et al., and Gülmez et al., which could be due to the higher susceptibility of the mitral valve to fluid overload and calcification [11, 12, 20, 34, 35]. In addition, our investigations revealed that right-sided heart valve vegetation, especially tricuspid valve vegetation, was significantly more frequent in the HD group than in the non-HD group. However, this result has not been previously described in similar investigations. Therefore, further studies are required to confirm and validate this finding. Simultaneous left and right-sided valve vegetations were more common in the HD patients, suggesting the aggressive nature of the disease in this group of patients. In addition, the mean vegetation size was significantly larger in HD versus non-HD patients (18.6 ± 11.8 mm vs. 12.1 ± 8.7 mm).

Studies by Kwon et al., Zhang et al., and Durante-Mangoni et al. could not demonstrate any significant differences in terms of vegetation of size and location between HD and non-HD patients [20, 24, 36].

### Outcomes and course of the disease

Similar to Rekik et al., we found a higher frequency of extracardiac embolism in the HD group [12]. Based on our results, only pulmonary artery embolism was significantly more common in the HD group. The fact that tricuspid valve vegetation was more frequent in the HD group may have caused this cohort’s higher prevalence of septic embolism in the pulmonary arteries. Nonetheless, in a recent study, Wang et al. reported no significant difference between HD and non-HD groups regarding embolic and neurological complications [27].

In our study, valve surgeries were performed on 52.94% of the HD patients, similar to the non-HD group (57.14%). In line with several previous investigations by Durante-Mangoni et al., Hsiao et al., and Kwon et al., there was no significant difference in the surgical requirements between the studied groups [24, 26, 36]. However, other studies by Stahl et al., Pericas et al., and Zhang et al. reported cardiac surgery being performed in a significantly lower number of HD patients than in non-HD patients [19, 20, 25]. Our study population was selected among patients referred to a tertiary cardiovascular center who may have required special treatment or been in a worse condition than those at primary or secondary centers. Hence, such differences between our findings and those of other studies are expected and can help understand all aspects of IE in different clinical settings.

Consistent with most prior studies, in-hospital mortality was significantly higher in the HD group than in the non-HD group [5, 19, 20, 24, 27]. However, Stahl et al. and Durante-Mangoni et al. reported no significant differences between the two groups [25, 36]. In addition, further analysis revealed that the rate of in-hospital mortality was significantly higher in surgically treated HD patients compared to surgically treated non-HD patients. This finding is in line with the result of a study by Raza et al. who reported 13% hospital mortality for HD patients versus 5% for non-HD patients following surgical intervention [37].

Moreover, within HD patients, in-hospital mortality rates were similar for medically versus surgically managed patients (43.8% vs. 38.9%). An investigation by Jones et al. reported numerically lower but statistically indistinguishable in-hospital mortality in HD patients with surgery (11.1% vs. 15.2%) [18]. A recent meta-analysis by Ting et al. examining surgery versus medical therapy specifically in HD infective endocarditis patients also found no difference in the survival outcomes between these interventions [38].

The mean length of hospital stay for HD patients was lower than that for non-HD patients. This is probably because of the higher mortality rate in the former group.

### Limitations

This study has several limitations worth acknowledging. First, the retrospective observational design based on registry data restricts our ability to infer causality between hemodialysis status and clinical outcomes. Second, our cohort was restricted to patients admitted to a single tertiary academic medical center. Results may not be generalizable to other healthcare settings or geographic regions. Third, longer-term follow-up data was not available in the Iranian Registry of Infective Endocarditis (IRIE), including details on readmissions, post-discharge mortality, other long-term outcomes, and specifics on surgical interventions performed. Thus, our data and analyses were limited by the constraints of the registry. Fourth, the sample size, while determined to be adequately powered for the primary outcome examined, remains relatively small overall and in the hemodialysis subgroup specifically. Larger multi-center prospective studies are warranted to validate findings.

## Conclusion

Our findings support the growing body of research showing a poor prognosis for infective endocarditis in the context of end-stage renal failure. Patients with HD exhibited a unique microbiological and clinical profile, with a higher incidence of culture-negative illness. In more than half of the cases, the mortality of HD patients remained disproportionately high even after surgery. These results highlight the need for further investigation to inform preventative strategies and best practices for managing this susceptible population. Strategies that facilitate early detection and treatment of HD patients before the onset of advanced disease may enhance prognosis. Our research thoroughly accounts for infective endocarditis in one of the largest Middle Eastern HD populations documented. More multicenter prospective studies in this area are necessary to clarify the exclusive epidemiological and medical aspects of IE in this region.

## Data Availability

All data gathered and analyzed during this study are included in this published article. In addition, the dataset used is available from the corresponding author on reasonable request.

## Abbreviations

AV: Aortic valve
CRP: C-reactive protein
E. coli: Escherichia coli
ESR: Erythrocyte sedimentation rate
ESRD: End-stage renal disease
HD: Hemodialysis
IE: Infective endocarditis
MRSA: Methicillin-resistant Staphylococcus aureus
MV: Mitral valve
PV: Pulmonary valve
S. aureus: Staphylococcus aureus
S. bovis: Streptococcus bovis
S. viridans: Streptococcus viridans
TV: Tricuspid valve
WBC: White blood cell
